# In-plane stimulated emission of polycrystalline CH_3_NH_3_PbBr_3_ perovskite thin films†

**DOI:** 10.1039/c9ra08619f

**Published:** 2020-01-15

**Authors:** Ju Wang, Wei Liu, Cuncun Wu, Ning Zhu, Congyue Liu, Shufeng Wang

**Affiliations:** State Key Laboratory for Artificial Microstructure and Mesoscopic Physics, Department of Physics, Peking University Beijing 100871 China wangsf@pku.edu.cn; Collaborative Innovation Center of Extreme Optics, Shanxi University Taiyuan Shanxi 030006 China; Frontiers Science Center for Nano-optoelectronics, Peking University Beijing 100871 China

## Abstract

Hybrid organic–inorganic lead halide perovskites have been investigated extensively within the last decades, for its great potential in efficient solar cells and as an ideal light source. Among the studies on stimulated emission (SE), the emission is either out-of-plane for polycrystalline films or in-plane with randomly aligned single microcrystals and nanowires. In this work, we revealed in-plane propagation of SE from bromine-based perovskite polycrystalline thin films (CH_3_NH_3_PbBr_3_, or MAPbBr_3_). The output from in-plane SE is an order higher than the out-of-plane emission. It is proposed that large crystalline flakes in the films lead to the in-plane lasing phenomena. The output coupling can be found at grain boundaries, intergrain gaps, and artificial structures. Simulative results support the experimental phenomenon that large crystalline grains are profitable for in-plane propagation and over 90% photons can be sufficiently outcoupled when the gap is larger than a micron. Considering the fabrication and handling convenience, we propose that the MAPbBr_3_ thin films can be easily integrated for in-plane applications as the light source for photonic chips *etc.*

## Abstract 2

MAPbBr_3_ perovskite thin film contains large crystal flakes, which support the in-plane stimulated emission and its propagation within these polycrystalline films. The emission scatters at the natural or artificial edge of the film.

## Graphical abstract

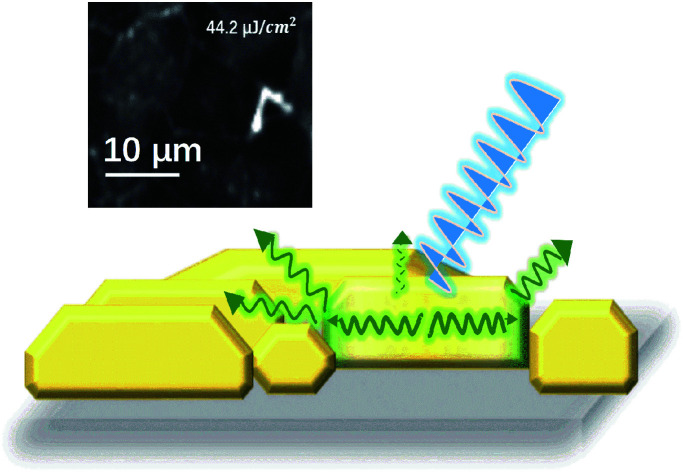

## Introduction

Organic–inorganic hybrid lead perovskites have emerged recently as promising materials for many applications in photovoltaics and optoelectronics. With remarkable properties such as effective light absorption,^1^ high photoluminescence quantum yield,^2^ long carrier lifetimes and diffusion lengths,^3^ its luminescent properties arouse much interest. These features make perovskite a novel material in luminescent devices of the light emitting diode (LED), semiconductor laser, high-resolution imaging, optical integral, optical coding and so on.^4–6^ To meet distinctive demands in the next generation of lighting equipment, researchers have created various structures of perovskite, such as nanoparticles, nanowires, nanoplates, photonic crystals, and metasurfaces.^7–9^ In most cases, perovskite can perform a non-linear optical gain and produce an emissive burst at a certain wavelength under intensive excitation. This phenomenon was firstly discovered as amplified spontaneous emission (ASE) in thin films.^10^ Lasing was also found in cavity structures like nanowires or nanoplates, where polaritons has been introduced to explain the gain mechanism in these strong-coupling regime.^11^ Moreover, it has been thought to be superfluorescence in the structure of perovskite superlattice.^12^ Abundant works had been done to explore the physical origin and performance enhancement of these artificial structures. However, the studies focused on randomly deposited single crystal nanostructures like nanowire and nanoplates,^13,14^ or polycrystalline thin films, both of which are hard to be used for two-dimensional photonic integration as the integrated light source, either because of the difficulty of *in situ* growing at desired sites, or for not in-plane emission, respectively. Noted by large microcrystal flakes with sizes over 10 μm in MAPbBr_3_ films and convenient fabrication in our former study,^15^ it is possible to explore in-plane SE with the polycrystalline thin films, which will undoubtedly help to advance the applications of perovskite materials and integrated photonics.

In this work, we used a home-made fluorescence microscope to study spatial and spectroscopic resolved MAPbBr_3_ thin films. A clear in-plane SE is discovered, with uniform and an order higher output, from the grain boundaries and artificial structures, than the out-of-plane output.^16,17^ It can be attributed to the microcavity within each large grain and close packing of the grains. Previous reports argued that a crystallite can support total internal reflecting and waveguiding.^18,19^ This work proves that the SE emanating from the inner part of a crystallite. It can be effectively amplified and waveguided, and finally outcoupled from the boundaries.^20,21^ Also, our experiment shows that the film boundary can be designed and manipulated. The findings provided a new way to generate in-plane SE from the polycrystalline MAPbBr_3_ thin films as the light source, which is proposed to stimulate perspective applications in photonic integration.

## Results and discussion

**Fig. 1:**
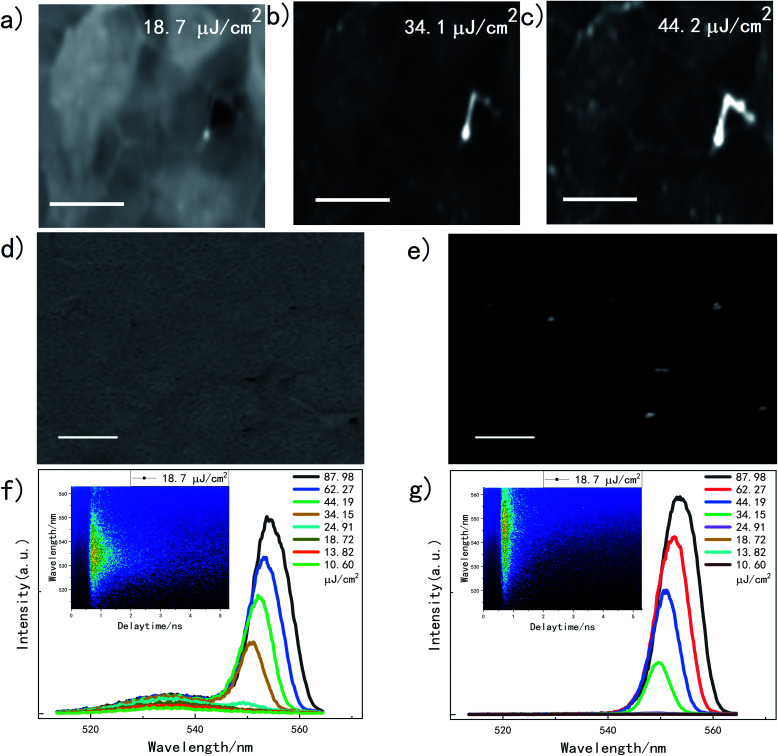
(a–c) Fluorescence under different excitation intensity, with scale bar of 10 μm. (d) SEM image of MAPbBr_3_ thin film. (e) Differential luminescence under excitation intensity of 34.1 μJ cm^−2^ and 18.7 μJ cm^−2^. (f) and (g) Spectra at the internal and boundary of the grains with increasing excitation intensity, respectively. Insets are the time-resolved fluorescence spectra collected by a steak camera.

Our samples were synthesized using the method of one-step spin-coating. To get a well crystallized structure, a lower saturated vapor pressure were proven beneficial.^22^ A favorable synthetic environment enables to create large grains averaging ∼10 μm with a quite flat surface (Fig. S1a†), which symbolizes a good crystallization. According to previous report,^15^ MAPbBr_3_ is the only member to form such large grains among all the widely-used perovskite composition, which makes it convenient to be observed under an optical microscope. A system combining a microscope and steak camera can provide direct visualization and accurate quantization of luminescence behavior of the sample spatially and temporally. The images collected by the microscope present localization dependent response to an increasing excitation intensity (Fig. 1a–c). At low excitation, uniform fluorescence within a microcrystal of the thin films can be seen, though brightness varies between the microcrystals (Fig. 1a). The boundaries start to stand out after a certain excitation threshold, indicating strong scattering of internal field at the edge of microcrystal (Fig. 1b and c). A video is composed to show the variation (ESI†). Fig. 1d presents the SEM image of the MAPbBr_3_ thin films, showing flatness of the film and some micron-sized vacancy between the grains. By subtracting fluorescent images at excitation of 34.1 cm^2^ and 18.7 cm^2^, we get the difference of the luminesce, shown in Fig. 1e, in which its bright spots fit well with the vacancies in SEM image of Fig. 1d. Fig. 1f shows the emission spectra of the internal of the gain. The fluorescence peak is around 535 nm, whose intensity increase without an obvious peak shift. The inset image on the top-left is the time-resolved fluorescence spectrum under the excitation of 18.7 cm^2^. Exceeding a certain excitation intensity, SE at 550 nm arises at a much faster growth, which was firstly discovered in 2014 and are widely known now.^10,23^

Similar spectral behavior at the boundary is shown in Fig. 1g. However, it is worth noting that the fluorescence at the wavelength of 535 nm is not apparent, while a peak at 550 nm with a much shorter lifetime is recorded and shown as inset of Fig. 1g. This is consistent to the previous reports,^24^ since lower energy peak comes from optically active defects, which is mainly due to the existence of excess PbBr_2_ at grain boundaries.^13,25^ From the spectrum in Fig. S1c,† compared with inside crystalline, an obvious red shift and a widen FWHM in SE at boundary are presented.

**Fig. 2:**
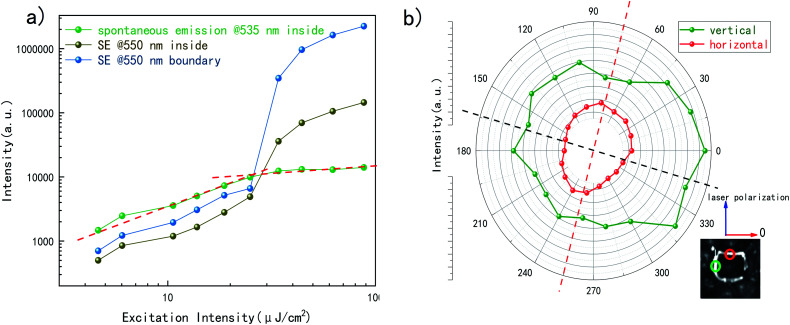
(a) Power dependence of fluorescence and SE at inside and boundary of a grain. (b) Polarization of orthogonal edges of a single grain.

Taking a look at the pump intensity dependent emission behavior at internal of the crystal grain, the fluorescence intensity at 535 nm are plotted in Fig. 2a. Below the threshold of 24 μJ cm^−2^, it is the spontaneous emission. All emissions at internal and boundary increase at a slope of ∼1. At this range the emission of 550 nm is part of fluorescence. According to our precious work,^26^ this slope reveals that radiance particles are mostly excitons. With the increasing of excitation intensity, SE at 550 nm increases superlinearly while the growth rate of fluorescence at 535 nm is restrained to 0.11, indicating a suppress in fluorescence when SE begins to show up. At higher pump intensity (about five times higher than threshold), the SE at internal and boundary become saturated and increases linearly to pump intensity. Both the increments of internal and boundary at 550 nm show a shape of ‘S’, which is typical in the lasing mechanism of semiconductors.^27^ The two turning points for both boundary and inside are nearly the same. However, the SE at the boundary bursts with significantly faster increment and finally an order brighter than the internal. The difference of increment rate of SE indicates that output of SE from boundary and inside are not of same process, *i.e.*, the in-plane light amplification is much more efficient than the out-of-plane SE. We also compare the relative ratio at boundary and inside (Fig. S1b†). Considering the luminescent flux reflects the collection of excited particles, the result shows that when excited above the threshold, about nine tenth of the SE photons tend to scatter from the boundaries.

Then the excitation polarization dependency should also be noticed. The excitation beam was set horizontal and parallel to the surface. Focusing on a single grain with orthogonal boundaries, the sample platform was rotated to realize a polarized excitation. Horizontal side is set to zero degree. Presented in Fig. 2b, excited at the intensity of 44.2 cm^2^, the red points show the SE intensity of one edge of the crystal while the green ones show the perpendicular edge. The red ones reach its relative maximum and minimum, while the green ones behave just the opposite way. A video in ESI† gives out a direct changing in emission intensity with different excitation polarization. Comparing with a clearly linear polarization dependency of a nanowire under the same experimental installation^28^ (see Fig. S2a†), this 0.2 polarization of a single grain is kind of weak. However, as the crystalline structures in thin film is intentionally aligned randomly, its insensitivity to the excitation polarization compensate the uncertainty of the crystal orientation and provide relative stable output as the light source, regardless of excitation polarization.

**Fig. 3:**
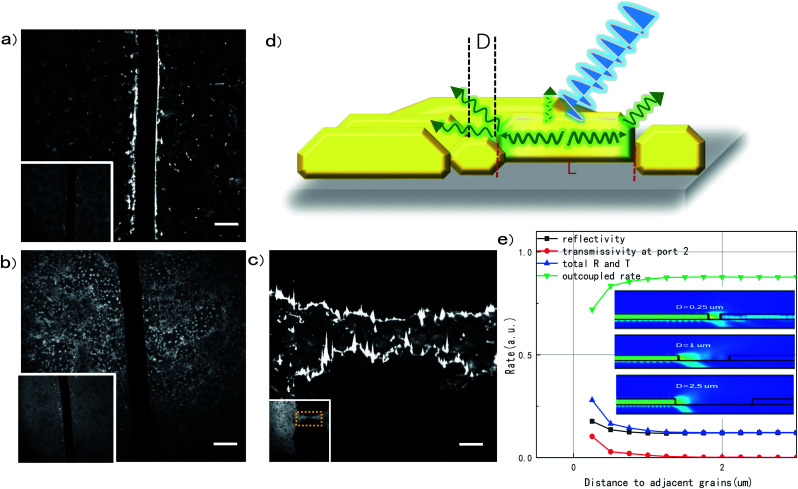
(a) and (b) Manually scratched lines in MAPbBr_3_ thin films with a large grain size (∼10 μm) and FAPbBr_3_ with a grain size around 1 μm under the excitation of 34.1 μJ cm^−2^, respectively. Inset is corresponding fluorescence imaging excited at 10.6 μJ cm^−2^, with scale bar of 10 μm. (c) Voids ablated by laser (800 nm) under high excitation. Inset is the same place amplified by 10× objective lens. (d) Scheme of the photon propagation within and at boundary of polycrystalline MAPbBr_3_ perovskite film. (e) Simulation results of SE outcouple at various grain intervals.

To prove the in-plane propagation of SE and reveal the possibility of manipulating the controllable output, detects are deliberately introduced to the films. Fig. 3a present a deep cut which we drew on the surface with a needle tip to produce artificial edges. The width of the groove is 10 μm, comparable to the size of the grain. The edges on both sides shine brightly and continuously when the film is excited above the threshold, indicating an effective photon accumulation. For a film with smaller grains, a shining edge with less brightness can also be seen (Fig. S3a†). Lifetimes in MAPbBr_3_ thin films with smaller grains are generally shorter than larger ones (Fig. S3c and d†), as more boundary leads to more defects in crystallization.^29,30^ It seems that smaller grains will reduce the in-plane SE. For other perovskite films, which contain only submicron crystal grains, no obvious in-plane SE can be found at these edges. *E.g.*, a FAPbBr_3_ thin films, as a control sample, has grains at the order of 1 μm or less (Fig. 3b). Due to the small size of grains, the SE behavior of FAPbBr_3_ seems to be random lasing^31^ (Fig. S4a–c†). SE in small-sized grains mainly scatters from voids and defects. They present a weaker emission at voids due to a shorter gain media. The ratio of in-plane and out-of-plane SE of FAPbBr_3_ is ∼4, while it is ∼15 in MAPbBr_3_. In addition, no SE photon accumulation can be found in artificial edges in these films, showing its difficulty to manipulate light emission in such films. In fact, crystal size, as well as its geometry, was found to play an important role in luminous properties.^32^

Besides the mechanical machining of grooves or natural defects for SE output, other processing methods can also be applied for producing a port as photon outlet. For example, laser is a powerful tool to surface portray.^33,34^ A series of voids, which were ablated by a high energy femtosecond laser amplifier with a wavelength of 800 nm, is presented in Fig. 3c. The edges are also bright due to the output of in-plane SE. The results affirm the feasibility of machining, which may make it easy to be proceeded as a light source at require location for photonic integration.

The SE at boundary and inside synchronously happens and shares an identical threshold, indicating they have the same excitation basis. However, the amplification at the boundary is an order higher than inside when excited above the threshold. This phenomenon shows that the in-plane SE experiences longer amplification path than the out-of-plane SE. The in-plane SE happened only at perovskite films with larger crystal grains of a few microns and vanished in films with small grains of less than a micron, indicating that the SE is not supposed to jump across the grain boundary, while the cavity resonance within a grain take a critical role for the amplification of SE. On the other hand, the in-plane SE shows red-shift of peak energy compared to out-of-plane SE demonstrated in SI (Fig. S1c†), which may come from the long distance propagation and band-edge absorption. We can also notice a slightly red shift in emission peak and spectrum broadening, which may result from weak diffusion and phonon collision when traveling through the inside of the crystallite.^20^

The weak polarization dependency at the edge of a single grain resembles the typical behavior of waveguide, which additionally proves that flakes in thin films are less effected by excitation source polarization comparing with structured devices such as nanowire. Based on experimental results, a sketch of emission mechanism is presented in Fig. 3d. It shows that SE arouses when the whole crystalline is excited above the thresholds. A small part of photons goes upward and scatters from the top. The others are amplified along the gain medium with a length of *L* and scatter massively from the naturally formed intervals with a width *D*. To understand the efficiency of SE output at the edge of grooves, a simulative results mimicking a modified one-dimensional propagation is demonstrated in Fig. 3e. When intervals exceed 1 μm, nine-tenth of photons will be outcoupled to outside of grains. This result matches well with experiments in Fig. 1e, which proves that the voids over 1 μm on continuous film can effectively scatter the SE. For inter-grain space less than 1 μm, a relatively small part of in-plane SE will be coupled to neighboring grains, as shown in Fig. 3d. Also, grain sizes greatly affect propagation properties, as boundaries are barriers to affect the in-plane propagation. Finally, the natural properties that halide perovskite can be advanced to flexible lasing devices will definitely broaden the material's prospects.^35,36^ Convenient fabrication and natural large grains make MAPbBr_3_ thin films more accessible, affordable, and operable for further uses as in-plane light source.

## Experimental

### MAPbBr_3_/FAPbBr_3_ thin film fabrication

Typical precursors (1 mM MABr/FABr and 1 mM PbBr_2_ dissolved in 1 mL DMSO) were deposited onto ITO substrates through flash evaporation technique. The solutions were spun on glass substrates at 4000 revolutions per minute for 6 s. And perovskite films were annealed on a hot plate (110 °C, 20 min) in a vacuum chamber (1.3 kPa, ∼10 s).

### Optical characterization

Samples are placed on a rotatable stage under the oblique incidence at an angle of 45°. We use a CCD, which is equipped with a Nikon camera lens, to record the real time image, while a steak camera (Hamamatsu C10910) is used to get the spectrum. Two modes are switched by simply adding a resettable reflector. The magnification of the objective lens is 50, so a clear resolution of micron-order structure is realized. Furthermore, a pinhole with a diameter of 200 μm at the image plane helps to distinguish the spectral behavior of an area around 4 μm. In our experiment, using the microscope and pinhole to locate a fixed point, we put on the reflector to get its spectral behavior.

### Simulations

Numerical simulations were performed using the COMSOL. The grain sizes were set at 10 μm and the thickness was set as 200 nm. The effect dielectric constant was 2.5. The reflectivity, transmissivity and loss ratio at the boundaries (named outcoupled rate) are calculated with intervals of 0.1 to 3 μm.

## Conclusion

In this work, we studied the in-plane SE of perovskite thin films. Using a fluorescence microscope, an intuitive mapping showed that the boundary and natural vacancy shined an order higher than the inside of the grains when it was excited above the threshold. Spectroscopic results convincingly demonstrated that photon can be amplified along the large grains in MAPbBr_3_ thin films and effectively scattered at boundaries. Stimulation results proved that micron-sized voids were sufficient for photons to scattering without coupling to neighbors. We also confirmed the possibility to manipulate the coupling behavior of the thin films simply by machining operation or by laser ablation. These results may help to reveal the physical essence of perovskite thin films SE, and may open up a new way in designing thin film devices for integrated photonics.

## Conflicts of interest

There are no conflicts to declare.

## Supplementary Material

RA-010-C9RA08619F-s001

RA-010-C9RA08619F-s002

RA-010-C9RA08619F-s003

## References

[cit1] De Wolf S., Holovsky J., Moon S.-J., Löper P., Niesen B., Ledinsky M., Haug F.-J., Yum J.-H., Ballif C. (2014). J. Phys. Chem. Lett..

[cit2] Deschler F., Price M., Pathak S., Klintberg L. E., Jarausch D.-D., Higler R., Hüttner S., Leijtens T., Stranks S. D., Snaith H. J., Atatüre M., Phillips R. T., Friend R. H. (2014). J. Phys. Chem. Lett..

[cit3] Shi D., Adinolfi V., Comin R., Yuan M., Alarousu E., Buin A., Chen Y., Hoogland S., Rothenberger A., Katsiev K., Losovyj Y., Zhang X., Dowben P. A., Mohammed O. F., Sargent E. H., Bakr O. M. (2015). Science.

[cit4] Service R. F. (2019). Science.

[cit5] Du W., Zhang S., Shi J., Chen J., Wu Z., Mi Y., Liu Z., Li Y., Sui X., Wang R., Qiu X., Wu T., Xiao Y., Zhang Q., Liu X. (2018). ACS Photonics.

[cit6] Fan Y., Wang Y., Zhang N., Sun W., Gao Y., Cheng-Wei Q., Song Q., Xiao S. (2019). Nat. Commun..

[cit7] Zhang C., Xiao S., Wang Y., Gao Y., Fan Y., Huang C., Zhang N., Yang W., Song Q. (2019). Laser Photonics Rev..

[cit8] Makarov S., Furasova A., Tiguntseva E., Hemmetter A., Berestennikov A., Pushkarev A., Zakhidov A., Kivshar Y. (2019). Adv. Opt. Mater..

[cit9] Hou S., Xie A., Xie Z., Tobing L. Y. M., Zhou J., Tjahjana L., Yu J., Hettiarachchi C., Zhang D., Dang C., Teo E. H. T., Birowosuto M. D., Wang H. (2019). ACS Photonics.

[cit10] Xing G., Mathews N., Lim S. S., Yantara N., Liu X., Sabba D., Grätzel M., Mhaisalkar S., Sum T. C. (2014). Nat. Mater..

[cit11] Zhang S., Shang Q., Du W., Shi J., Wu Z., Mi Y., Chen J., Liu F., Li Y., Liu M., Zhang Q., Liu X. (2018). Adv. Opt. Mater..

[cit12] Rainò G., Becker M. A., Bodnarchuk M. I., Mahrt R. F., Kovalenko M. V., Stöferle T. (2018). Nature.

[cit13] Fang X., Zhang K., Li Y., Yao L., Zhang Y., Wang Y., Zhai W., Tao L., Du H., Ran G. (2016). Appl. Phys.
Lett..

[cit14] Wang Z., Liu J., Xu Z.-Q., Xue Y., Jiang L., Song J., Huang F., Wang Y., Zhong Y. L., Zhang Y., Cheng Y.-B., Bao Q. (2016). Nanoscale.

[cit15] Liu Y., Wang J., Zhu N., Liu W., Wu C., Liu C., Xiao L., Chen Z., Wang S. (2019). Opt. Lett..

[cit16] Wu X., Jiang X.-F., Hu X., Zhang D.-F., Li S., Yao X., Liu W., Yip H.-L., Tang Z., Xu Q.-H. (2019). Nanoscale.

[cit17] Stranks S. D., Wood S. M., Wojciechowski K., Deschler F., Saliba M., Khandelwal H., Patel J. B., Elston S. J., Herz L. M., Johnston M. B., Schenning A. P. H. J., Debije M. G., Riede M. K., Morris S. M., Snaith H. J. (2015). Nano Lett..

[cit18] Pazos-Outón L. M., Szumilo M., Lamboll R., Richter J. M., Crespo-Quesada M., Abdi-Jalebi M., Beeson H. J., Vrućinić M., Alsari M., Snaith H. J., Ehrler B., Friend R. H., Deschler F. (2016). Science.

[cit19] Liu X., Zhang Q., Xiong Q., Sum T. C. (2013). Nano Lett..

[cit20] Mohan V., Jain P. K. (2017). J. Phys. Chem. C.

[cit21] Fang Y., Wei H., Dong Q., Huang J. (2017). Nat. Commun..

[cit22] Xiao Z., Dong Q., Bi C., Shao Y., Yuan Y., Huang J. (2014). Adv. Mater..

[cit23] Yakunin S., Protesescu L., Krieg F., Bodnarchuk M. I., Nedelcu G., Humer M., De Luca G., Fiebig M., Heiss W., Kovalenko M. V. (2015). Nat. Commun..

[cit24] Li W., Long R., Tang J., Prezhdo O. V. (2019). J. Phys. Chem. Lett..

[cit25] Shan D., Tong G., Cao Y., Tang M., Xu J., Yu L., Chen K. (2019). Nanoscale Res. Lett..

[cit26] Wang W., Li Y., Wang X., Lv Y., Wang S., Wang K., Shi Y., Xiao L., Chen Z., Gong Q. (2016). Phys. Rev. B.

[cit27] Eaton S. W., Lai M., Gibson N. A., Wong A. B., Dou L., Ma J., Wang L.-W., Leone S. R., Yang P. (2016). Proc. Natl. Acad. Sci. U. S. A..

[cit28] Wang X., Shoaib M., Wang X., Zhang X., He M., Luo Z., Zheng W., Li H., Yang T., Zhu X., Ma L., Pan A. (2018). ACS Nano.

[cit29] Kirakosyan A., Chinh N. D., Sihn M. R., Jeon M.-G., Jeong J.-R., Kim D., Jang J. H., Choi J. (2019). J. Phys. Chem. Lett..

[cit30] Wetzelaer G.-J. A. H., Scheepers M., Sempere A. M., Momblona C., Ávila J., Bolink H. J. (2015). Adv. Mater..

[cit31] Liu S., Sun W., Li J., Gu Z., Wang K., Xiao S., Song Q. (2016). Opt. Eng..

[cit32] Li B., Zhou T., Fang X., Zhang W., Li X., Guan Z., Li J., Wang L., Hark S., Zhang Z. (2019). J. Mater. Chem. C.

[cit33] Zhou C., Cao G., Gan Z., Ou Q., Chen W., Bao Q., Jia B., Wen X. (2019). ACS Appl. Mater. Interfaces.

[cit34] Chou S. S., Swartzentruber B. S., Janish M. T., Meyer K. C., Biedermann L. B., Okur S., Burckel D. B., Carter C. B., Kaehr B. (2016). J. Phys. Chem. Lett..

[cit35] Wu W., Wang X., Han X., Yang Z., Gao G., Zhang Y., Hu J., Tan Y., Pan A., Pan C. (2019). Adv. Mater..

[cit36] Wang Y.-C., Li H., Hong Y.-H., Hong K.-B., Chen F.-C., Hsu C.-H., Lee R.-K., Conti C., Kao T. S., Lu T.-C. (2019). ACS Nano.

