# Supplementary material for: In-plane stimulated emission of polycrystalline CH3NH3PbBr3 perovskite thin films
Source: RSC Adv. 2020 Jan 15;10(5):2703–8. doi: 10.1039/c9ra08619f (PMC9048420; doi:10.1039/c9ra08619f)
Supplement: RA-010-C9RA08619F-s003 [file RA-010-C9RA08619F-s003.pdf]

Electronic Supplementary Material (ESI) for RSC Advances.
This journal is © The Royal Society of Chemistry 2019

In-plane stimulated emission of polycrystalline $CH_3NH_3PbBr_3$ perovskite thin films

Ju Wang,[a] Wei Liu,[a] Cuncun Wu,[a] Ning Zhu,[a] Congyue Liu,[a] Shufeng Wang,*[a,b,c]

List

Videos

Images of MAPbBr_3 thin films under various excitation intensity

Images of MAPbBr_3 thin films under different excitation polarization

Additional figures

Figure S1
Figure S2
Figure S3
Figure S4

[a] *State Key Laboratory for Artificial Microstructure and Mesoscopic Physics, Department of Physics, Peking University, Beijing 100871, China*

[b] *Collaborative Innovation Center of Extreme Optics, Shanxi University, Taiyuan, Shanxi 030006, China*

[c] *Frontiers Science Center for Nano-optoelectronics, Peking University, Beijing 100871, China*

† Electronic Supplementary Information (ESI) available: graphs and videos to verify spectroscopic properties of different sample.

Additional figures

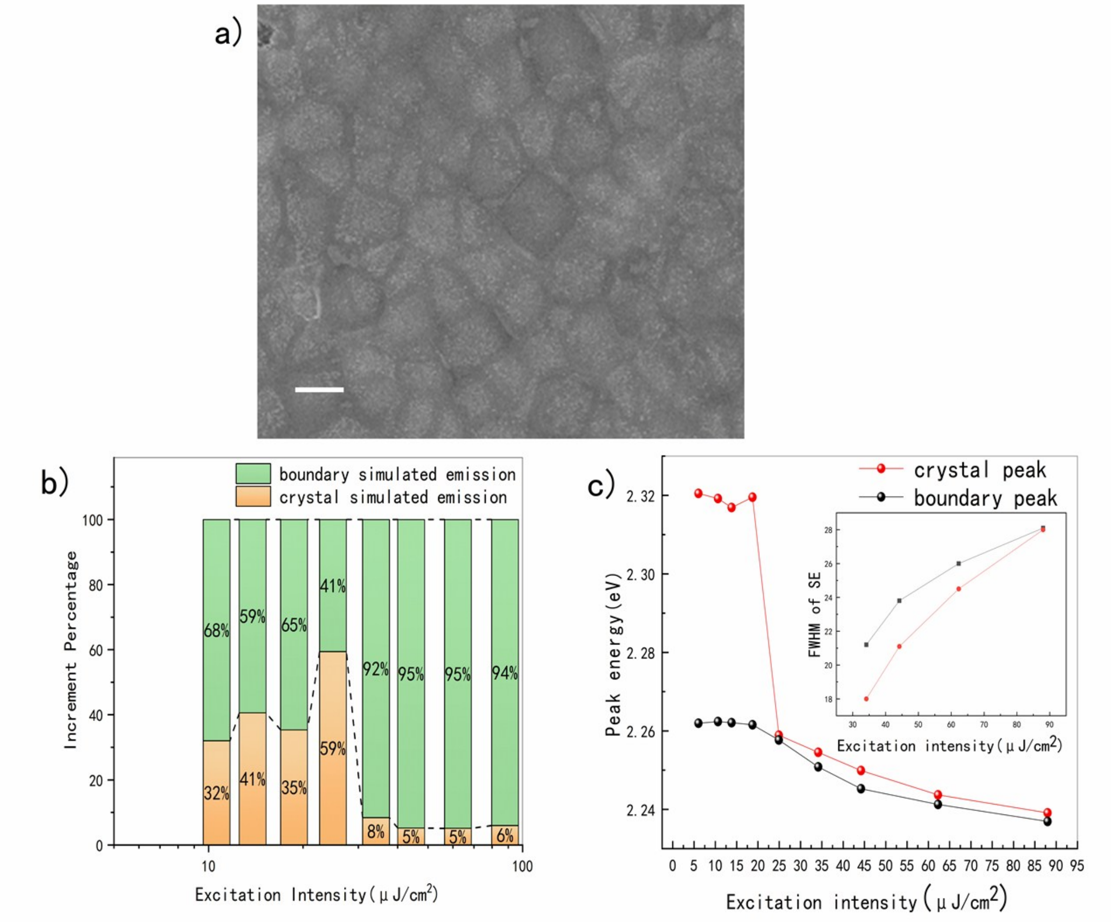

Figure S1. (a) SEM images of MAPbBr_3 thin film. Scalebar 10 µm. (b) SE intensity increment at boundaries and inside grains. (c) Peak energy and FWHM of inside grains and boundaries.

SEM image shows the grains in MAPbBr_3 thin films averages about 10 μm, and boundaries are quite dim due to the tightly arrangement of grains. We compare the SE increment at boundary (green) and inside (yellow). Considering the luminescent flux reflects the collection of excited particles, the result shows nine tenth of newly added stimulated emission tend to gather and scatter from boundaries when excited above the threshold. Also, SE emissions at boundaries is red-shift and broadened a little in peak energy and FWHM compared with inside of crystal.

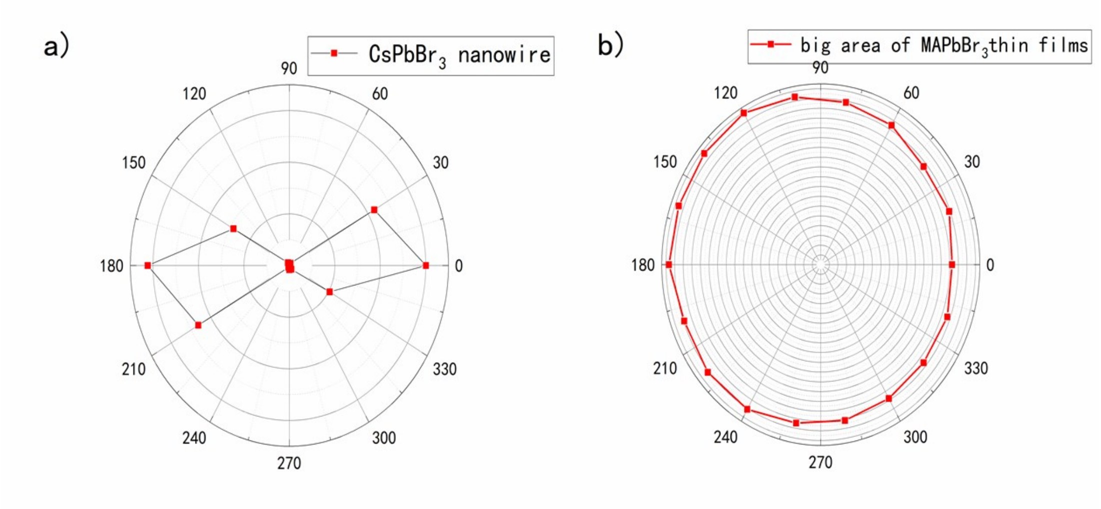

Figure S2. (a) Polarization of a nanowire under 44.2 $\mu J/cm^2$ excitation. (b) Polarization of large area of MAPbBr_3 thin films under 44.2 $\mu J/cm^2$ excitation.

CsPbBr_3 nanowires show a clearly linear polarization under 44.2 $\mu J/cm^2$ excitation, which means its emission will be greatly modulated by the excitation polarization. Using the same system, a large area of 160 µm in MAPb Br_3 thin films shows no polarization, since a large cluster of grains and polycrystalline have no uniform orientation.

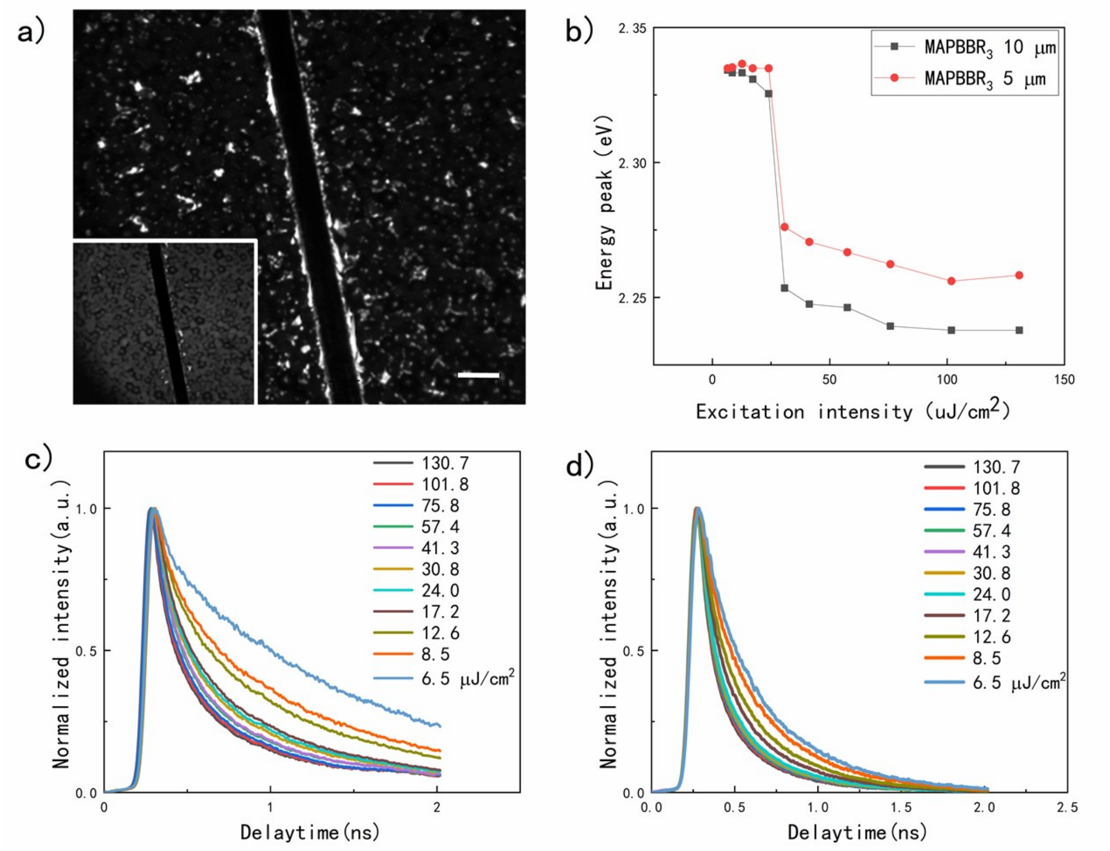

Figure S3. (a) Emission behavior in MAPbBr_3 thin films with 5 μm grains. Scalebar 10 μm. (b) Peak energy under various excitation. (c), (d) Fluorescence lifetimes in MAPbBr_3 thin films with 10 μm and 5 μm grains.

A shining edge can also be seen in MAPbBr_3 thin films with smaller grains (5 μm). but less bright compared to the larger ones. A blue-shifted peak energy and a shortened lifetime of SE are also observed.

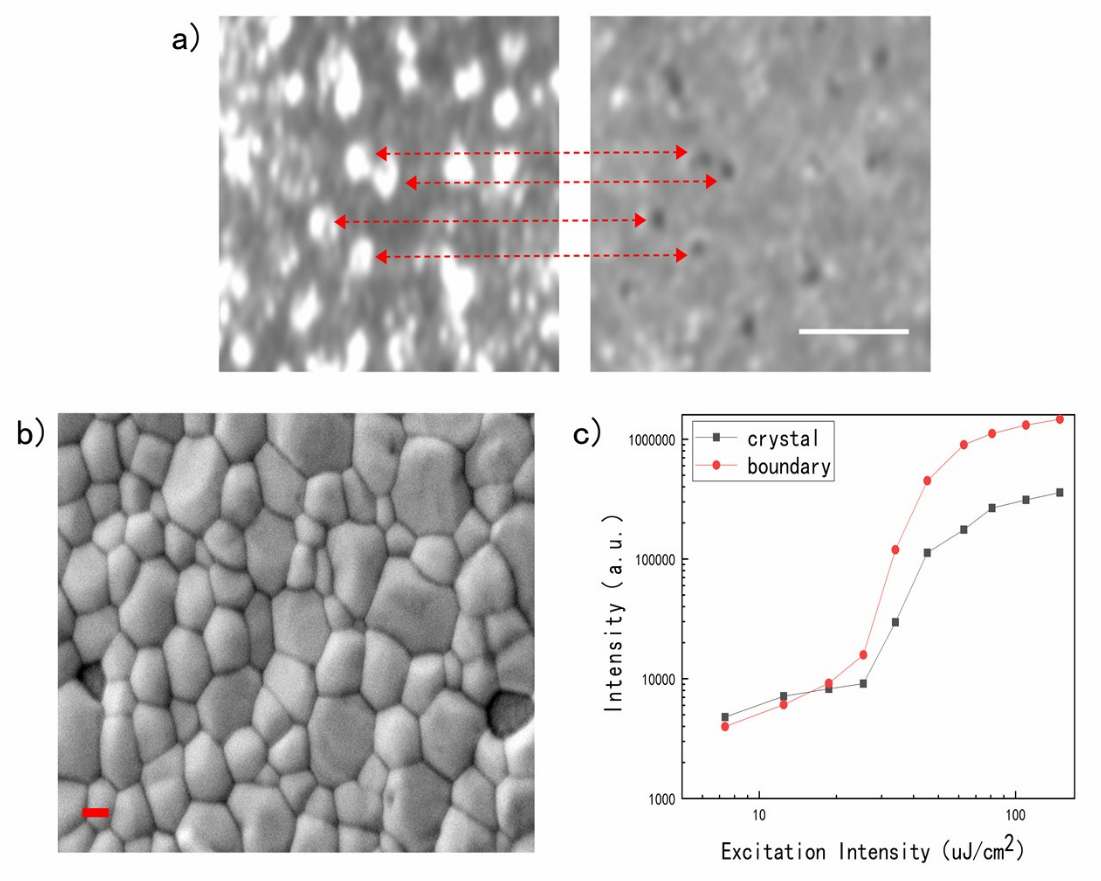

Figure S4. (a) Fluorescence image of FAPbBr_3 thin films under the excitation of 8.09 $\mu J/cm^2$ (left) and 12.4 $\mu J/cm^2$ (right). Scalebar 10 µm. (b) SEM image of FAPbBr_3 thin films. Scalebar 200 nm. (c) Power law relations at the inner part and viods.

Fluorescence below and above threshold SEM images of FAPbBr_3 thin films shows that SE scatter a lot from naturally formed voids and defects. SEM image presents a clear crystalline of 1 µm and below. Power law using the same set-up shows that emission at voids and inside shows a smaller gap, which can be attributed to the absence of effective propagation and amplification of SE.
